# Four self-made free surface electrospinning devices for high-throughput preparation of high-quality nanofibers

**DOI:** 10.3762/bjnano.10.218

**Published:** 2019-11-15

**Authors:** Yue Fang, Lan Xu

**Affiliations:** 1 National Engineering Laboratory for Modern Silk, College of Textile and Engineering, Soochow University, 199 Ren-ai Road, Suzhou 215123, China

**Keywords:** electric field, free surface electrospinning, high-throughput preparation, Maxwell 3D, mechanism, nanofibers

## Abstract

Four different self-made free surface electrospinning (FSE) techniques, namely, modified bubble-electrospinning (MBE), modified free surface electrospinning (MFSE), oblique section free surface electrospinning (OSFSE) and spherical section free surface electrospinning (SSFSE), designed for high-throughput preparation of high-quality nanofibers, are presented in this paper. The mechanisms of fiber preparation of the corresponding four FSE devices were studied by simulating the electric field distribution using the Maxwell 3D software. The properties of the electric field in the device are very important for the FSE process. The effects of the particular technique on the morphology and the yield of nanofibers were experimentally investigated. The experimental data agree well with the results of the simulations and show that all four FSE devices can be used to prepare large quantities of high-quality nanofibers. A comparison of the spinning mechanisms of these four FSE devices illustrates that the SSFSE device performs best, providing the highest quality and yield of nanofibers. The SSFE device could yield 20.03 g/h of nanofibers at an applied voltage of 40 kV.

## Introduction

Due to their excellent properties, such as high surface-to-volume ratio and high porosity, nanomaterials have become more and more important in industrial manufacturing. As one of the most important methods for preparing nanomaterials, electrospinning (ES) [1–3] has received much attention [4–7]. However, with the advancement of nanotechnology and the increasing performance requirements of nanomaterials, the fatal shortcoming of the traditional ES process, its low yield [8–9], has also received more attention. Many studies have focused on improving the production efficiency of the ES technique. Ding et al. [10] electrospun nanofibers using a multiple-jet ES system. Krishnamoorthy et al. [11] demonstrated an ES setup consisting of 24 (8 × 3) nozzles for the large-scale production of aligned ceramic nanofibers. Kim et al. [12] developed an upward high-speed cylinder-type ES system with 120 needles in each cylinder in order to accomplish the mass production of nanofibers. In spite of the high production efficiencies reached, some problems during these ES processes still remain, such as the blocking of needles and interactions between jets.

Accordingly, in recent years, many needleless electrospinning methods were presented to obtain the high-throughput production of nanofibers [13–23]. Niu et al. [13] prepared high-throughput polyacrylonitrile (PAN) nanofibers using a needleless electrospinning method. He et al. [14–15] presented bubble electrospinning (BE) as an effective method for preparing nanofibers in batches. Thoppey et al. [16] successfully prepared poly(ethylene oxide) (PEO) nanofibers using a modified ES with a bowl collector. Jiang et al. [17] reached mass preparation of PAN nanofibers by a free surface ES technique. Wu et al. [18] studied high-throughput tipless electrospinning via a circular cylindrical electrode. Shin et al. [19] used a multiple vertical rod setup for needleless ES to fabricate submicrometer polymer fibers. Moon et al. developed a syringeless electrospinning technique with a helically probed rotating cylinder for preparing a nanofiber web [20]. The spinning parameters and yields of the established ES techniques are compared in Table 1.

**Table 1:** The spinning parameters and yields of the established electrospinning (ES) techniques. Modified bubble electrospinning (MBE); bubble electrospinning (BE).

ES method	Fiber material	Spinning voltage	Yield	Fiber diameter

single needle ES [8–9]	polyvinylpyrrolidone (PVP)	15–60 kV	0.01–0.1 g/h	75–1500 nm
multi-nozzle ES [11]	PVP	15 kV	several times of single needle ES	50–100 nm
coil ES [13]	polyacrylonitrile (PAN)	60 kV	10–23 g/h	200–400 nm
BE [15]	polyvinyl alcohol (PVA)	35 kV	3 g/h	–
bowl ES [16]	poly(ethylene oxide) (PEO)	16 kV	0.684 g/h	243–293 nm
free surface ES [17]	PAN	70 kV	100 times of single needle ES	488–576 nm
tipless ES [18]	PEO	50–80 kV	260 times of single needle ES	207–453 nm
needleless vertical rods ES [19]	PVA	30–50 kV	0.36–1.92 g/h	357–383 nm
ES with a helically probed cylinder [20]	PAN	15–17 kV	3.2 g/h	2–800 µm
MBE [21]	PVA	30–70 kV	19.8–72 g/h	108–168 nm
MBE [23]	silk fibroin (SF)	50 kV	3.1 g/h	50–313 nm

**Figure 1:**
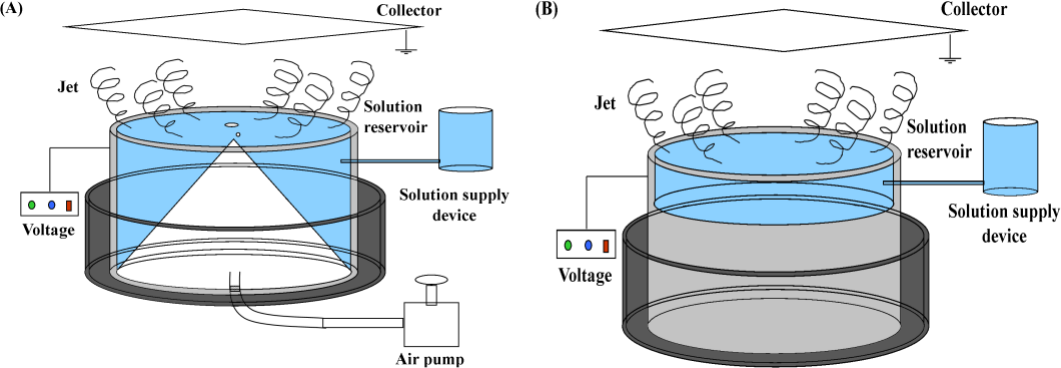
Schematic of (A) the modified bubble electrospinning (MBE) [21] and (B) the free surface electrospinning (FSE) (without air pump) devices.

In our previous work [21–22], a modified bubble electrospinning (MBE) method was proposed to fabricate high-quality PAN nanofibers at high yield. Moreover, we found that the addition of sodium dodecyl benzene sulfonate (SDBS) could significantly reduce the surface tension of the spinning solution facilitating the spinning process [24]. The mass production of silk fibroin nanofibers was successfully accomplished by this method [23]. A schematic of the MBE device is illustrated in Figure 1A.

As shown in Figure 1A, the MBE device mainly consists of a solution reservoir, a variable high-voltage power generator, an air pump, a collector and a solution supply device. Moreover, the solution reservoir is the main spinning part of the MBE device and includes a copper pipe and a cone-shaped polymer nozzle. Based on the MBE device, three additional self-made devices used for so-called modified free surface electrospinning (MFSE), oblique section free surface electrospinning (OSFSE) and spherical section free surface electrospinning (SSFSE) are introduced in this paper to obtain high-throughput preparation of high-quality nanofibers through modifications of the solution reservoirs. In contrast to the MBE device, the three other FSE devices have no air pump, as illustrated in Figure 1B, and thus only apply electric forces to form jets on the surface of the spinning solution by overcoming its surface tension.

The effects of the MBE, MFSE, OSFSE and SSFSE device design on the morphology and the yield of the produced nanofibers were experimentally investigated. The differences between them were explained based on simulations of the electric field distribution using the Maxwell 3D software. Our results show that all four FSE devices can be used to prepare large quantities of high-quality nanofibers, whereas the SSFSE aparatus is the optimal FSE device, providing the highest quality and the highest yield of nanofibers.

## Experimental

### Materials

Polyacrylonitrile (PAN, *M*_W_ = 150,000) was purchased from Beijing Lark Branch Co., Ltd. (Beijing, China). SDBS was purchased from Sinopharm Chemical Reagent Co., Ltd. (Shanghai, China). *N*,*N*-Dimethylformamide (DMF) was provided by Shanghai Chemical Reagent Co. Ltd. (Shanghai, China). The spinning solution was obtained by dissolving 10 wt % PAN and 1 wt % SDBS in DMF under magnetic stirring at 60 °C for 4 h using a thermostatic magnetic stirrer (DF-101S, Xinrui Instrument Factory, Changzhou, China) to yield a transparent liquid.

### Apparatuses

**Figure 2:**
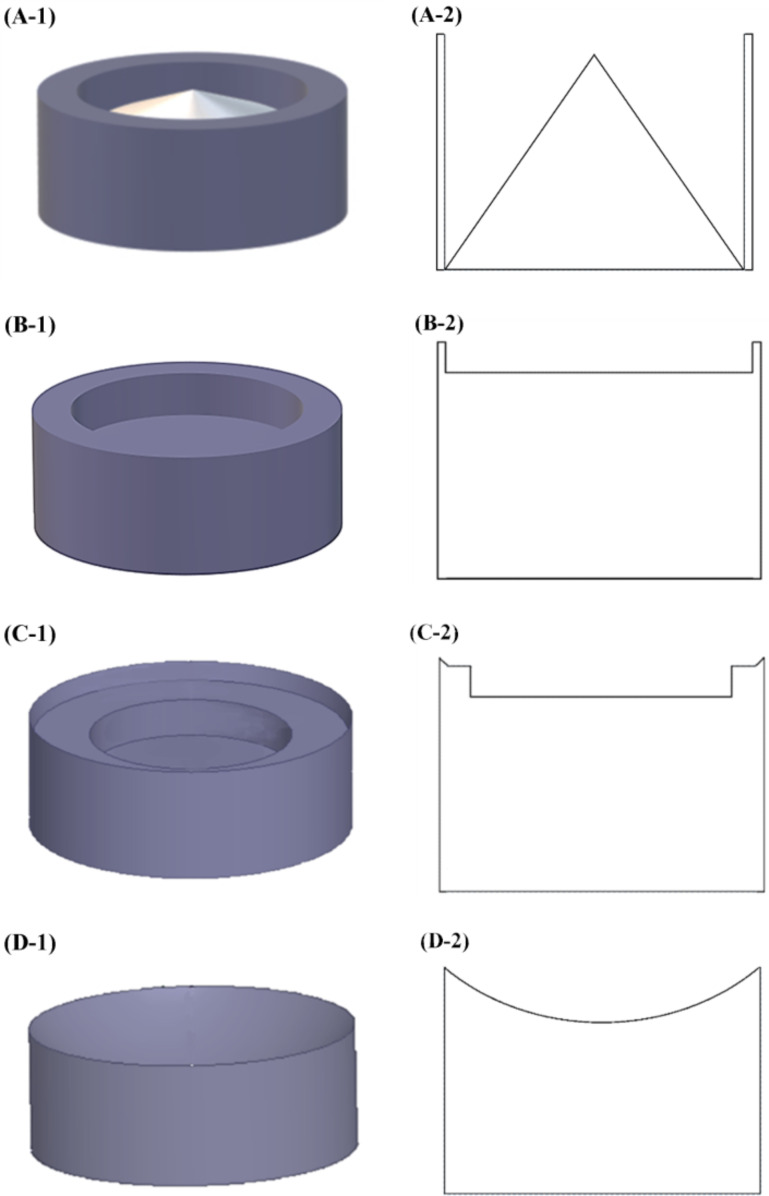
Schematic diagrams of the four solution reservoirs. (A-1), (B-1), (C-1) and (D-1) are the 3D schematic diagrams of the MBE, MFSE, OSFSE and SSFSE device, respectively. (A-2), (B-2), (C-2) and (D-2) are the corresponding longitudinal cross-sectional views.

Four kinds of FSE apparatuses with different solution reservoirs, namely MBE, MFSE, OSFSE and SSFSE, were designed and built in-house. Based on the MBE apparatus [14], as illustrated in Figure 1B, the other three apparatuses all consist of a self-made copper solution reservoir, a grounded collector over the reservoir and a variable high-voltage power generator (0–150 kV, TRC2020, Dalian Teslaman Technology Co., LTD), whose positive terminal is directly connected to the solution reservoir. Schematic diagrams of the four solution reservoirs are shown in Figure 2.

The solution reservoir of the MBE device is a copper cylinder with a height of 30 mm, an outer diameter of 40 mm and a wall thickness of 2 mm that contains a nylon cone-shaped air nozzle with a height of 28 mm and a bottom diameter of 36 mm. The solution reservoir of the MFSE device is a similar copper cylinder with a height of 30 mm and an outer diameter of 40 mm, in which a cylindrical groove with a radius of 18 mm and a height of 5 mm is dug from its upper surface. The solution reservoir of the OSFSE device is similar to that of the MFSE device, but the groove is made up of a circular truncated cone with a height of 2 mm, an upper bottom diameter of 40 mm and a lower bottom diameter of 36 mm, as well as a cylinder with a height of 8 mm and a diameter of 32 mm (Figure 2 (C-2)). The solution reservoir of the SSFSE apparatus is a copper cylinder with a height of 30 mm and an outer diameter of 40 mm that is truncated by a ball with a radius of 50 mm.

### Self-made free surface electrospinning (FSE) devices

According to our previous works [21–23] and other studies [17,19], it was found that as the voltage increases, the diameter of the nanofibers becomes smaller, the distribution of the nanofibers is more uniform, and the yield of the nanofibers increases. However, an excessive voltage can lead to the instability of the electric field, and moreover, it involves safety risks that would be fatal in industry. Therefore, in this study, a low spinning voltage of 40 kV was chosen to study the spinning performance of the four self-made FSE devices, and the other spinning parameters were set as follows: the working distance from the solution reservoir to the grounded collector was 18 cm, and the collector surfaces (200 mm × 200 mm) were covered with conductive aluminum foil in order to easily remove the electrospun nanofibers for further measurements. The electrospun nanofibers were dried at room temperature and sputter-coated with a gold film for SEM analysis. All FSE experiments were carried out at room temperature (20 °C) and at a relative humidity of 60%.

**Figure 3:**
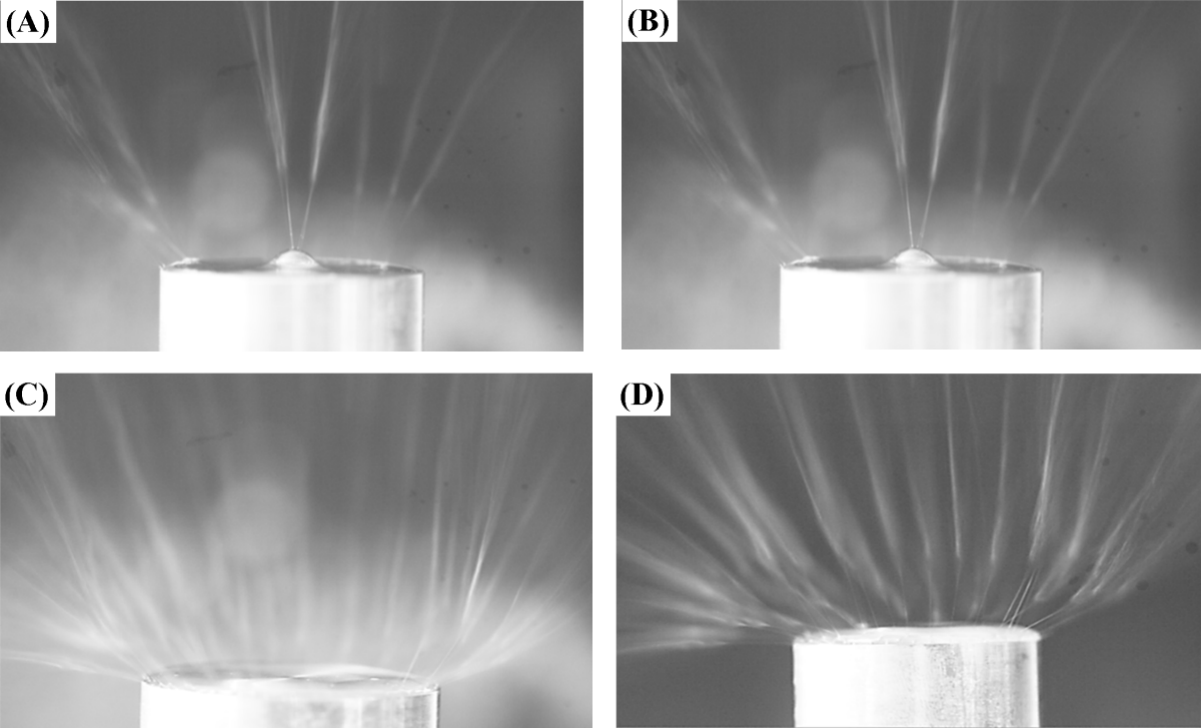
Photographs of the MBE (A), MFSE (B), OSFSE (C) and SSFSE (D) process.

**Figure 4:**
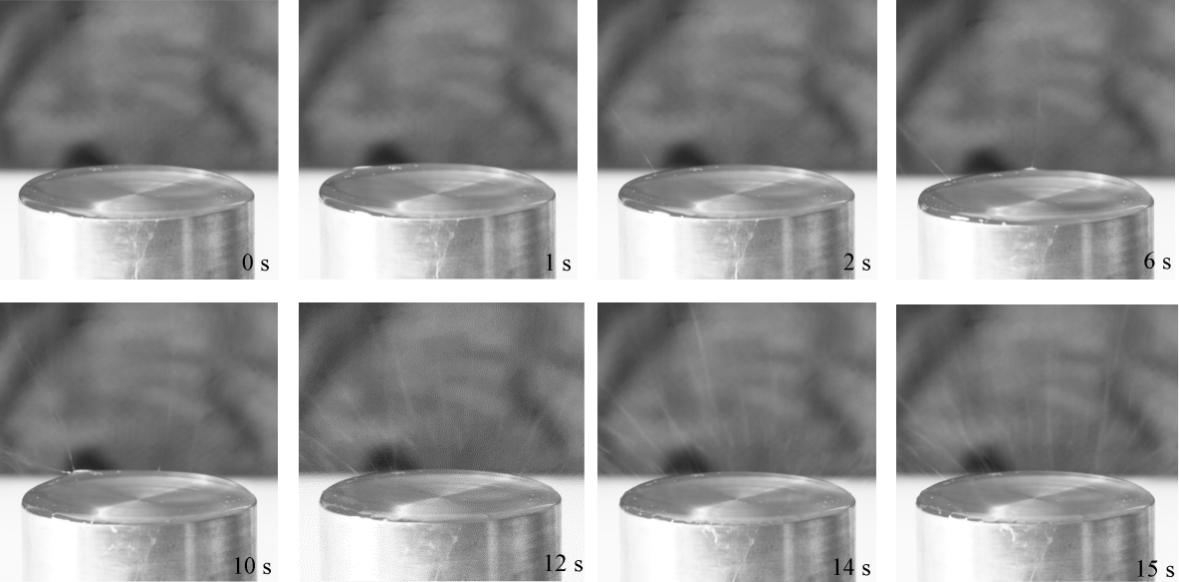
Time-lapse images (0–15 s) of the jet initiation process of OSFSE. The voltage was turned on and held at an amplitude of 40 kV throughout the spinning process.

**Figure 5:**
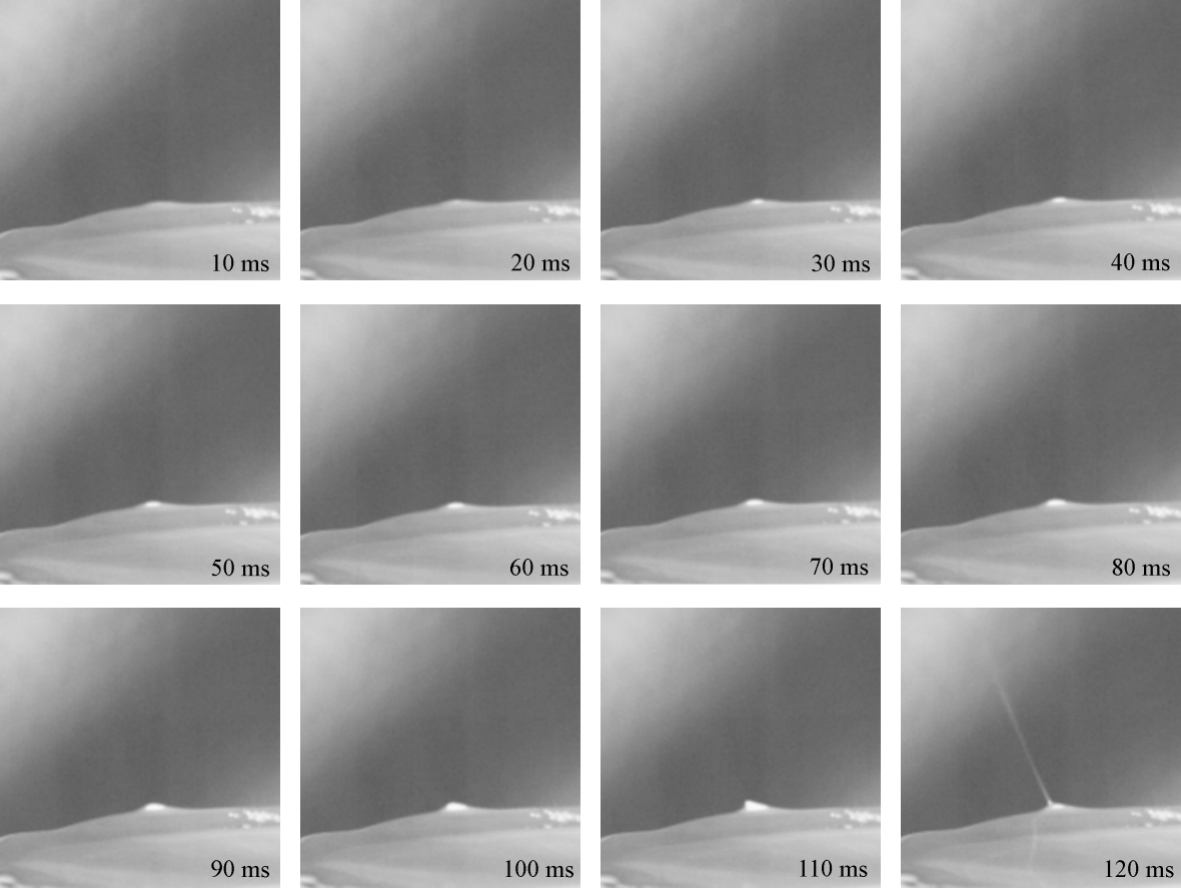
Time-lapse images (10–120 ms) of the jet initiation process of OSFSE. The voltage was turned on and held at an amplitude of 40 kV throughout the spinning process.

The spinning processes of the different devices were recorded using a high-speed camera at a frame rate of 100 frames/s (VRI-Phantom-VEO-L, Ametek, California, USA), as shown in Figure 3, Figure 4 and Figure 5. It can be seen that the multiple jets generated in the MBE process are concentrated primarily near the top edge of the copper reservoir and the top of the bubble. The multiple jets produced in the MFSE process are mainly concentrated near the top edge of the reservoir, while the multiple jets generated in the OSFSE and SSFSE processes appear on the entire solution surface. Comparing these photographs of the FSE processes, it is obvious that the number of jets produced in the MBE process is minimal, and the number of jets generated in the OSFSE and SSFSE processes is maximal.

The jet initiation in the OSFSE process viewed from the side by a high-speed camera is shown in Figure 4. Immediately after a voltage of 40 kV (which is above the threshold voltage) is applied to the solution surface, a deformation of the fluid is observed at the top edge of the solution reservoir. A Taylor-cone-like protrusion forms a jet after 2 s. Within 12 s, further jets rapidly develop starting at the reservoir top edge, finally covering the entire solution surface. After 15 s, the OSFSE process stabilizes.

Figure 5 shows further snapshots of the jet formation in the OSFSE process, taken as a side view by a high-speed camera. In the first 10–80 ms, the fluid at the top edge of the solution reservoir is pulled upward by the applied electric field. During the 80–110 ms interval, the fluid elongates, sharpens and forms a Taylor cone-like protrusion. Finally, at 120 ms, a jet forms.

**Figure 6:**
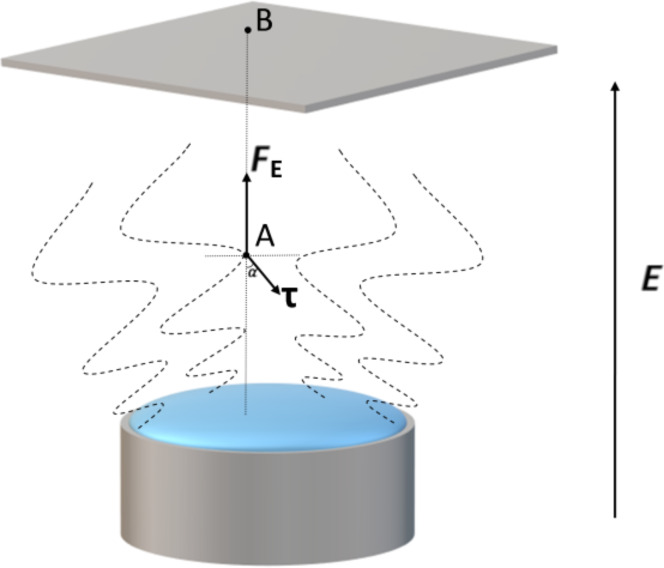
The forces acting at point A of the jet in the FSE process.

Figure 4 and Figure 5 illustrate the working principle of the free surface electrospinning method: the surface tension of the spinning solution is overcome by the applied electric field to form jets at the solution surface that finally touch the collector being stretched into nanofibers. Figure 6 shows the forces a solution particle experiences at point A on one of the jets produced in the FSE process when an electric field is applied between the solution reservoir and the collector, neglecting airflow resistance and any environmental interference. According to Figure 6, the horizontal component the of viscous force *F*_1_ produces a centripetal force resulting in the shrinking of the radius of the whipping circle. The resulting vertical force component *F*_2_ provides the kinetic energy of the jet, impelling the stretch of the jet towards the copper mesh collector. The force components are calculated as follows:

[1] $$F_{1}=\tau \sin\alpha =\left(av+bv^{2}\right)\sin\alpha$$

[2] $$F_{2}=F_{\text{E}}-\tau \cos\alpha =q_{\text{A}}E-\left(av+bv^{2}\right)\cos\alpha$$

where *v* is the velocity of the jet, *a* and *b* are constants to be further determined theoretically or experimentally, *q*_A_ is the signed magnitude of the charge at point A that is determined by the nature of the spinning solution, and *E* is the electric field intensity that is calculated using the applied voltage and the working distance.

According to the above equations and figures, the distribution of the electric field intensity plays a very important role in the FSE process since multiple jets form on the entire spinning area. To optimize the electric field distribution while keeping the applied voltage constant, the solution reservoir of the FSE apparatus was modified yielding the MBE, MFSE, OSFSE and SSFSE devices. It is necessary to simulate the distribution of the electric field intensity in the different FSE processes to illustrate and compare their spinning performance.

### Simulation of electric field

The distribution of the electric field between the solution reservoir and the collector in the four different self-made FSE devices was simulated using the Maxwell 3D software. The simulations were carried out using the following experimentally realized parameters: the copper reservoir as positive pole was a cylinder with a diameter of 40 mm and a height of 30 mm, the bulk conductivity of copper was set to 5.8 × 10^11^ µs/cm, the electric conductivity of the polymer nozzles in the MBE was 0 µs/cm, the electric conductivity of the PAN-DMF solution with a concentration of 10 wt % PAN and 1 wt % SDBS was 2372 µs/cm, the working distance was 180 mm and the applied voltage was 40 kV.

### Characterization

The morphology of the PAN nanofibers was characterized by scanning electron microscopy (SEM) (Hitachi S4800, Hitachi, Tokyo, Japan). The matrix morphology and the diameter of the fibers were characterized using the Image J software (National Institute of Mental Health, Bethesda, MD, USA) to study 100 randomly chosen nanofibers in 50 SEM images. The mass of the PAN nanofibers was measured as follows using a precise electronic balance (XJ120A, Precisa, Shanghai, China):

[3] $$W=\left(W_{1}-W_{0}\right)/t$$

where *W* is the yield of nanofibers, *W*_0_ and *W*_1_ are the weights of the aluminum foils before and after spinning and *t* is the spinning time.

## Results and Discussion

### Modeling the electric ﬁeld

**Figure 7:**
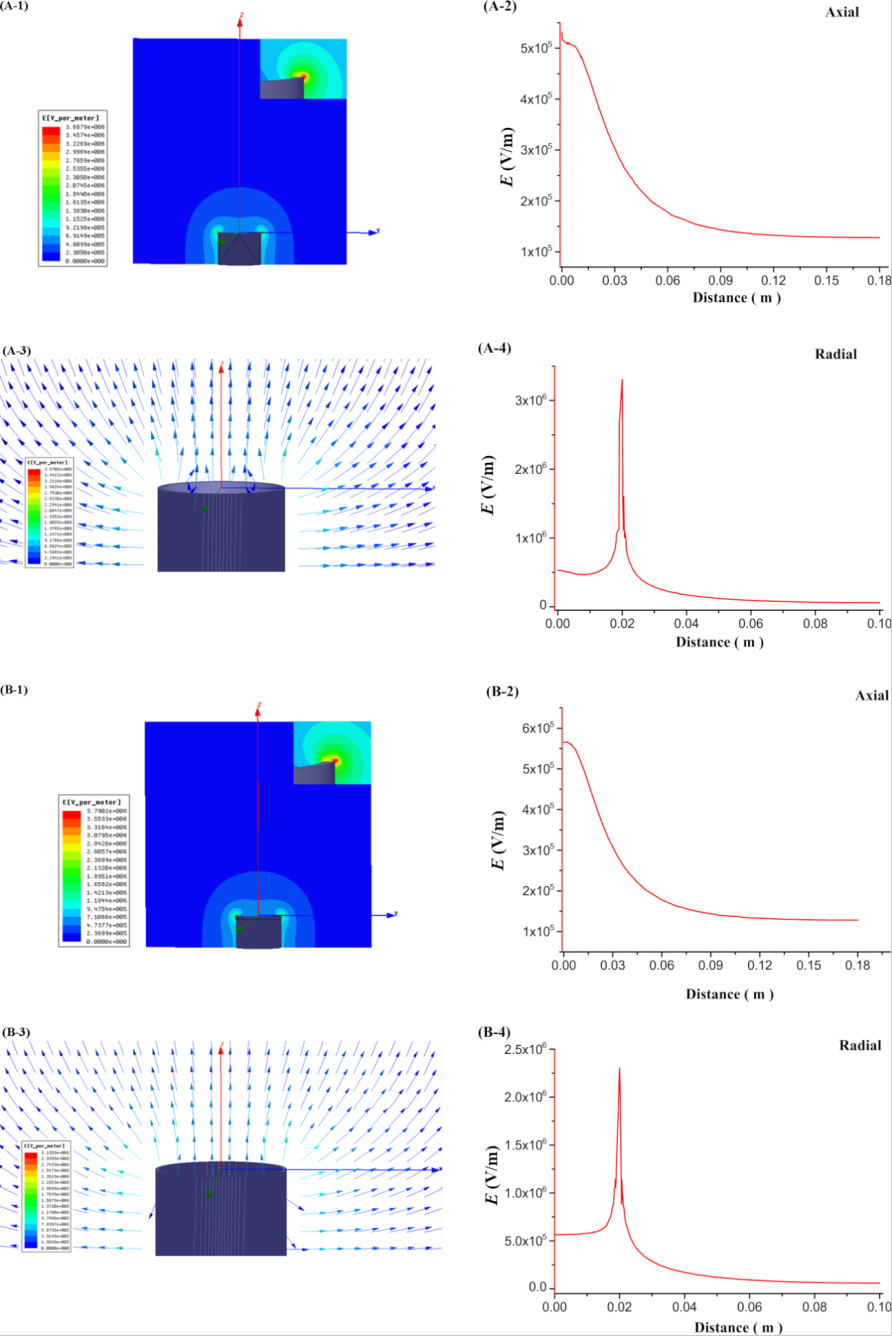
Simulation of the electric field in the MBE (A) and MFSE (B) devices.

**Figure 8:**
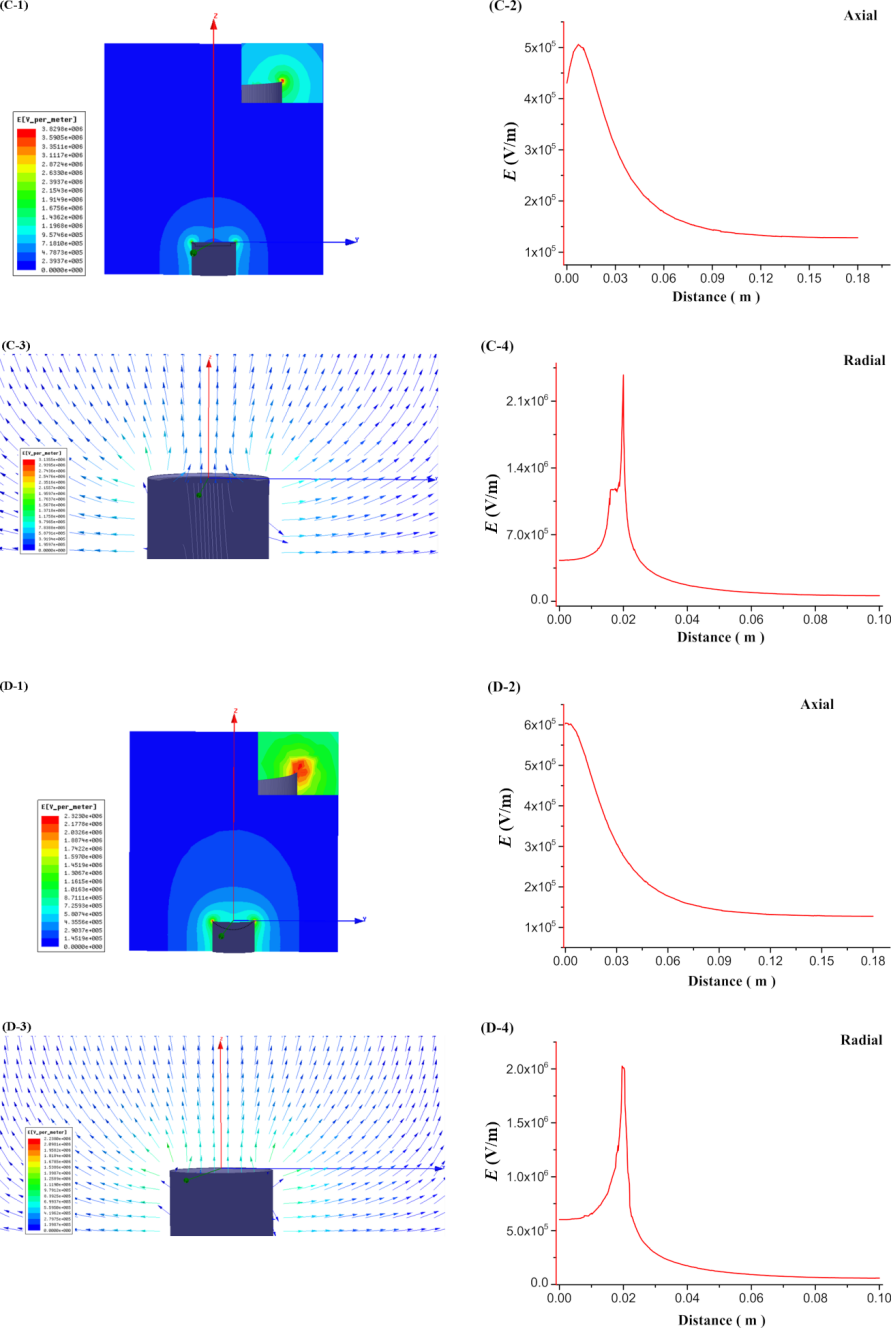
Simulation of the electric field in the OSFSE (C) and SSFSE (D) devices.

The results of the simulations of the electric field inside the MBE, MFSE, OSFSE and SSFSE devices are illustrated in Figure 7 and Figure 8. Figure 7 (A-1 and B-1) and Figure 8 (C-1 and D-1) show the scalar plots of the two-dimensional center sections of the 3D electric field simulations. The inset shows the magnification of the reservoir top edge. The colors illustrate the magnitude of the electric field, and the color legends are given at the left side. It is found that when a voltage of 40 kV is applied to the devices, the electric field maximum appears at the top edge of the reservoir. Figure 7 (A-3 and B-3) and Figure 8 (C-3 and D-3) are plots of the corresponding electric field vectors in the same region. The arrow color indicates the magnitude of the electric field. The vector plots demonstrate that the electric field at the solution surface directly points towards the collector, which is explained by the cylindrical symmetry of the reservoir leading to a cancellation of the vertical field components. In the case of the MBE device, the electric field vectors are relatively disordered, while for the SSFSE device, they are the mostly ordered. Figure 7 (A-2, B-2, A-4 and B-4) and Figure 8 (C-2, D-2, C-4 and D-4) display the shape of the axial (0–180 mm) and radial (0–100 mm) electric field components as a function of the distance from the solution surface at the center of the reservoir. These plots clearly reveal the maximum electric field positions and the corresponding intensity values. The axial distribution curves show that the electric field intensity decreases with increasing distance from the solution surface. The radial distribution curves reveal a sharp peak of the electric field intensities near the top edge of the reservoir resulting from the transition from copper to air. In contrast to the three other FSE devices, due to the influence of the polymer nozzle, the radial electric field intensity of the MBE device decreases to near zero before it increases slightly at larger distances.

To further compare the spatial distribution of the electric fields of the MBE, MFSE, OSFSE and SSFSE devices, a parameter ƒ is introduced as follows:

[4] $$f=\frac{E_{\max}}{E_{\text{av}}}$$

where *E*_max_ is the maximum electric field intensity and *E*_av_ is the average electric field intensity.

**Figure 9:**
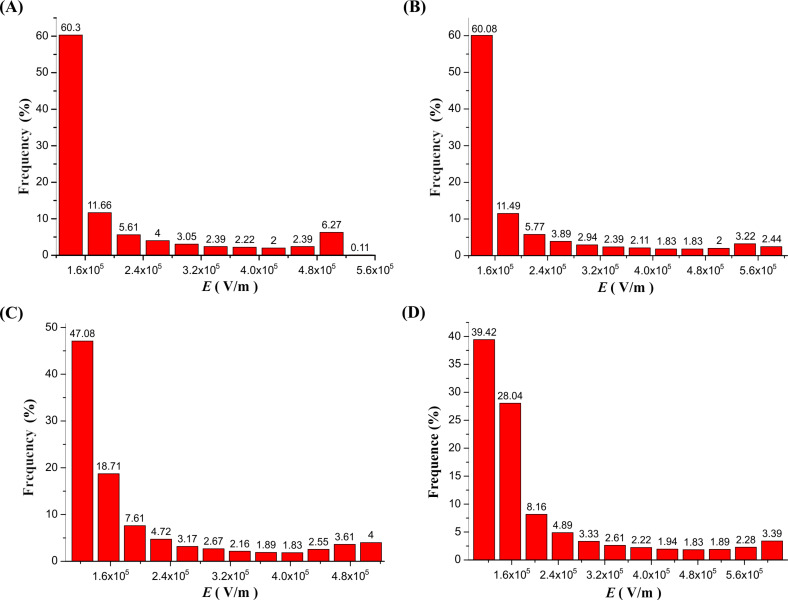
Distribution of the electric field along the vertical axis from the center of the solution surface to the collector (0–180 mm). A, B, C and D correspond to the MBE, MFSE, OSFSE and SSFSE device.

Figure 9 shows the distribution of the electric field along the vertical axis from the center of the solution surface to the collector (0–180 mm) for all four self-made FSE devices. The calculated values of *E*_max_, *E*_av_ and ƒ are listed in Table 2. There is little difference between the axial *f*-values of the four FSE devices. Hence, the axial distributions of the electric field are similar for the four FSE processes. The *E*_max_ values are the largest for the SSFSE device.

**Table 2:** Calculated values of *E*_max_, *E*_av_ and ƒ along the center axis (0–180 mm) of the four self-made FSE devices.

FSE process	*E*_max_ (V/m)	*E*_av_ (V/m)	*f*

MBE	5.31 × 10^5^	2.02 × 10^5^	2.62
MFSE	5.66 × 10^5^	2.07 × 10^5^	2.75
OSFSE	5.06 × 10^5^	2.02 × 10^5^	2.52
SSFSE	6.04 × 10^5^	2.08 × 10^5^	2.88

**Figure 10:**
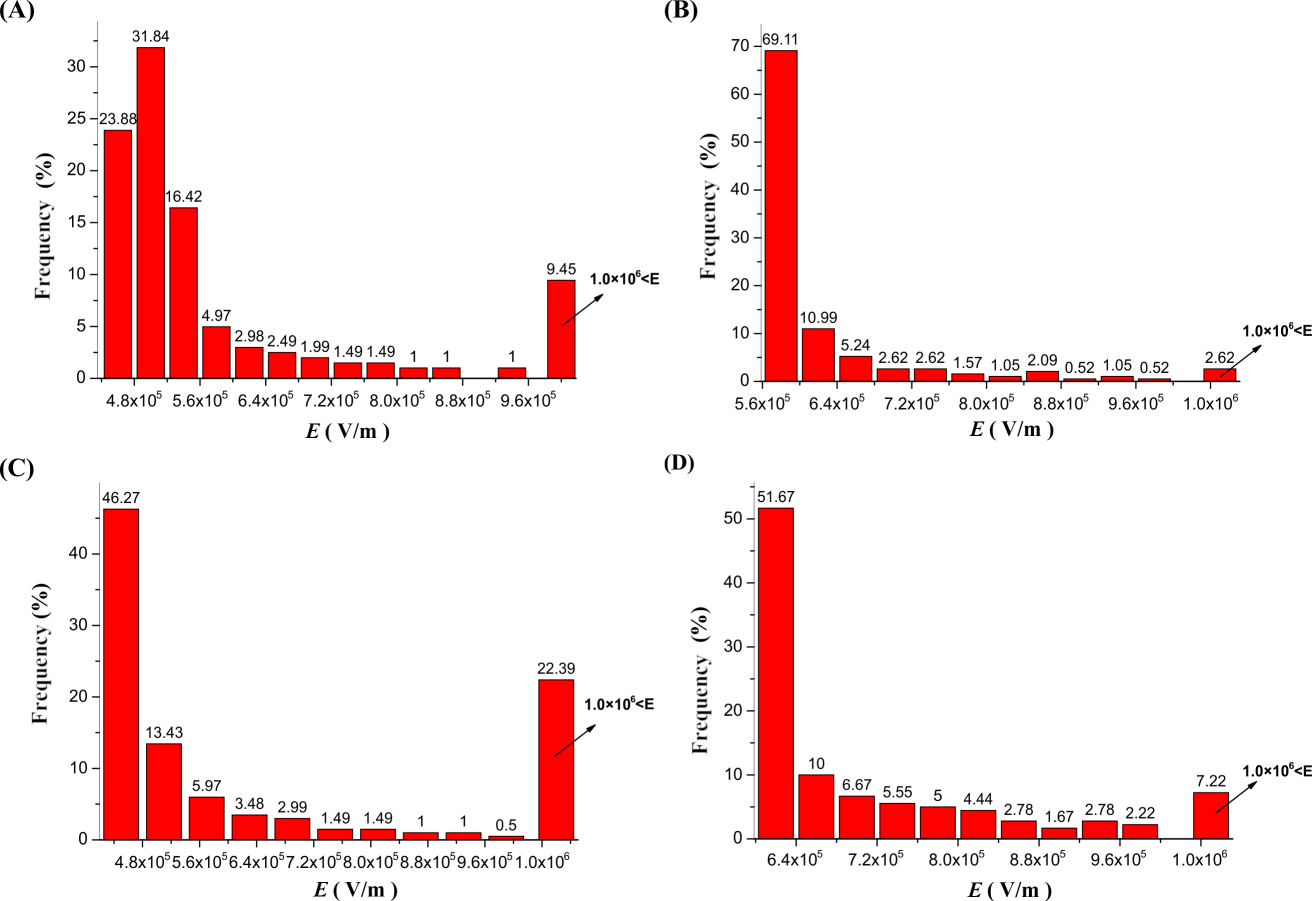
Distribution of the electric field along the radial axis (0–20 mm). A, B, C and D correspond to the MBE, MFSE, OSFSE and SSFSE device.

Figure 10 shows the distribution of the electric field along the radial axis (0–20 mm) from the center of the solution surface to the top edge of the reservoir. The calculated values of *E*_max_, *E*_av_ and ƒ are are given in Table 3. Comparing the four devices, Figure 9 reveals that the radial distributions of the electric field are quite different. To compare this difference in more detail, Table 3 gives a detailed overview of the intensity distribution of electric field (*E*_R_) along the radial axis (0–20 mm) of the four self-made FSE devices.

**Table 3:** Detailed distributions of the electric field intensity along the radial axis (0–20 mm) of the four self-made FSE devices.

*E*_R_ (V/m)	Frequency (%)			
	MBE	MFSE	OSFSE	SSFSE

4.0 × 10^5^ < *E*_R_ ≤ 4.4 × 10^5^	0.00	0.00	24.88	0.00
4.4 × 10^5^ < *E*_R_ ≤ 4.8 × 10^5^	0.00	0.00	21.39	0.00
4.8 × 10^5^ < *E*_R_ ≤ 5.2 × 10^5^	23.88	0.00	9.95	0.00
5.2 × 10^5^ < *E*_R_ ≤ 5.6 × 10^5^	31.84	0.00	5.97	0.00
5.6 × 10^5^ < *E*_R_ ≤ 6.0 × 10^5^	16.42	69.11	3.48	0.00
6.0 × 10^5^ < *E*_R_ ≤ 6.4 × 10^5^	4.98	10.99	2.49	51.67
6.4 × 10^5^ < *E*_R_ ≤ 6.8 × 10^5^	2.99	5.24	1.99	10.00
6.8 × 10^5^ < *E*_R_ ≤ 7.2 × 10^5^	2.49	2.62	1.99	6.67
7.2 × 10^5^ < *E*_R_ ≤ 7.6 × 10^5^	1.99	2.62	1.00	5.55
7.6 × 10^5^ < *E*_R_ ≤ 8.0 × 10^5^	1.49	1.57	1.00	5.00
8.0 × 10^5^ < *E*_R_ ≤ 8.4 × 10^5^	1.49	1.05	1.00	4.44
8.4 × 10^5^ < *E*_R_ ≤ 8.8 × 10^5^	1.00	2.09	0.50	2.78
8.8 × 10^5^ < *E*_R_ ≤ 9.2 × 10^5^	1.00	0.52	1.00	1.67
9.2 × 10^5^ < *E*_R_ ≤ 9.6 × 10^5^	0.00	1.05	0.50	2.78
9.6 × 10^5^ < *E*_R_ ≤ 1.0 × 10^6^	1.00	0.52	0.50	2.22
1.0 × 10^6^ < *E*_R_	9.50	2.62	22.39	7.22
*E*_max_	3.31 × 10^6^	1.14 × 10^6^	2.38 × 10^6^	2.03 × 10^6^
*E*_av_	6.82 × 10^5^	6.20 × 10^5^	6.90 × 10^5^	8.00 × 10^5^
*F*	4.84	1.77	3.48	2.50

From Figure 10 and Table 3, it can be found that the *f*-value of the MBE device is the largest, while that of the MFSE device is the smallest. This means that the electric field distribution inside the MBE device is the least uniform due to the influence of the polymer nozzle. In fact, most of the jets develop mainly on top of the bubble produced by the nozzle and near the top edge of the copper reservoir, as shown in Figure 3A. The electric field distribution of the MFSE is the most uniform showing no tip discharge phenomena. However, the values of the electric field intensities are the smallest, such that the multiple jets are primarily produced near the top edge of the reservoir, as illustrated in Figure 3B. The distinct edges of the reservoir of the OSFSE device (see Figure 2 (C-1 and C-2)) lead to a nonuniform electric field distribution, however with high electric field intensities. Multiple jets are generated on the entire solution surface, as shown in Figure 3C. Compared to the other FSE devices, the *f*-value of the SSFSE apparatus is relatively small, and its electric field intensities are the largest. Hence, multiple jets are produced on the entire solution surface, as displayed in Figure 3D. The smooth spherical shape of the reservoir makes the electric field distribution more uniform, while the tip discharge phenomenon appearing on the arc edge of the reservoir (see Figure 2 (D-1 and D-2)) maximizes the electric field intensity. These results show that the electric field simulations can be used to explain the FSE processes observed by the high-speed camera. We find that the SSFSE apparatus is the optimal FSE device characterized by ordered electric field vectors, a uniform electric field distribution and the largest electric field intensity, which will result in high-quality and high yield of nanofibers.

### Fiber morphology

**Figure 11:**
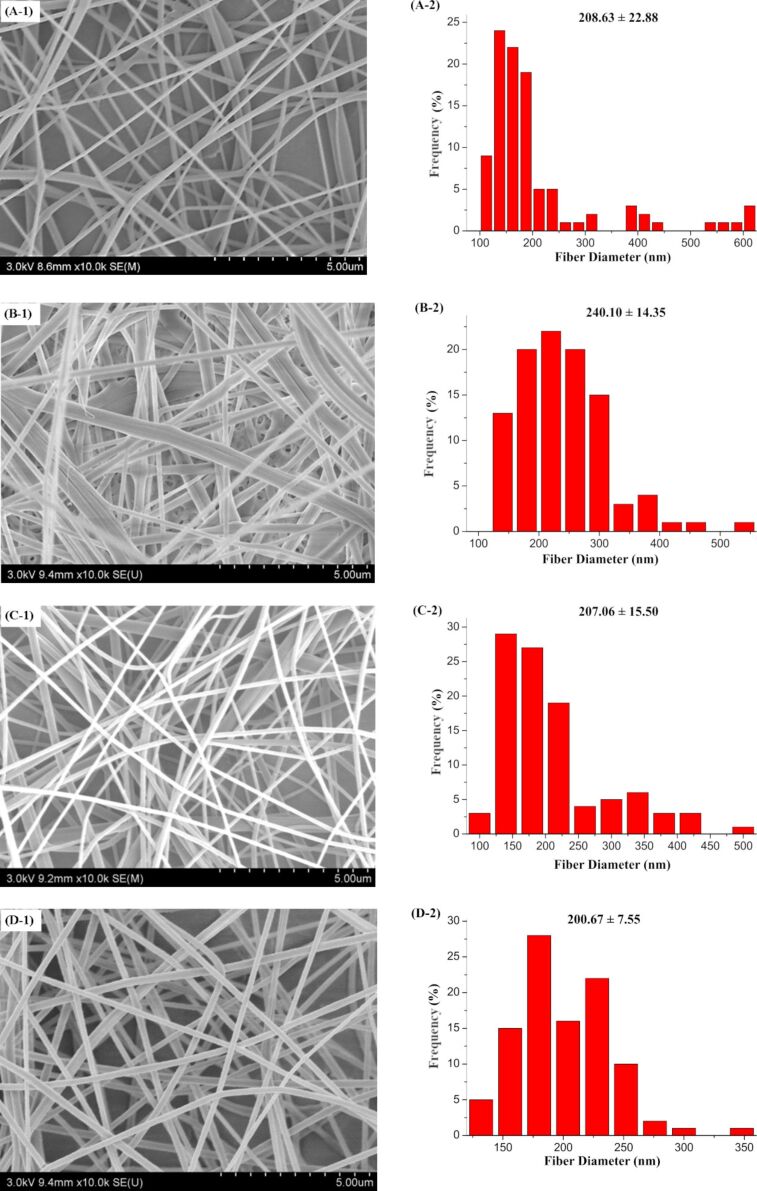
SEM images and the corresponding diameter distribution of the PAN nanofibers obtained by the four self-made FSE devices. A, B, C, and D correspond to MBE, MFSE, OSFSE and SSFSE.

The morphology of the PAN nanofibers produced by the four self-made FSE devices was investigated by SEM. Figure 11 shows the SEM images and the corresponding diameter distributions of the PAN nanofibers. The average diameters of the fabricated nanofibers along with the corresponding standard deviations and confidence intervals are presented in Table 4. The standard deviations are high as a result of measuring the nanofiber diameters by observing sample data. The given confidence intervals estimate the ranges in which diameters of nanofibers could still be observed. The estimated range is calculated from a given set of sample data [25].

**Table 4:** Average diameter of the nanofibers produced by the four different FSE devices.

FSE device	Average diameter (nm)	Standard deviation (nm)	Confidence interval (nm)

MBE	208.66	116.72	22.88
MFSE	240.06	73.21	14.35
OSFSE	207.06	79.08	15.5
SSFSE	200.67	38.52	7.55

The results from Figure 11 and Table 4 show that the average diameter of the nanofibers prepared by the MFSE method are the largest. The average diameters of the nanofibers obtained by the MBE, OSFSE, and SSFSE methods are similar to each other. However, it can be seen from Table 4 that the diameter distributions of the nanofibers produced by MFSE and SSFSE are more uniform as indicated by the smaller confidence intervals. The observed trend of the diameter variation is related to the previous results from the electric field simulations. Figure 7, Figure 8 and Table 3, show that the electric field intensity inside the MFSE device is the smallest. According to Equation 2, the larger the electric field intensity, the larger the electric force, which stretches the jet making the nanofiber diameter smaller. Therefore, the diameter of the nanofibers prepared by the MFSE method is the largest due to the smallest electric field intensity. In addition, the smaller the *f*-value of the FSE device, the more uniform the electric field distribution, resulting in a more uniform diameter distribution of the nanofibers. Indeed, the diameter distribution of the nanofibers produced by the MFSE and SSFSE methods is more uniform as a result of the corresponding smaller *f*-value. The quality of the nanofibers prepared by the MBE, MFSE, OSFSE and SSFSE methods was compared to each other, and it could be found that the quality of the nanofibers obtained by the SSFSE approch was highest, in line with the results of the electric field simulation.

### Yield of PAN nanofibers

**Figure 12:**
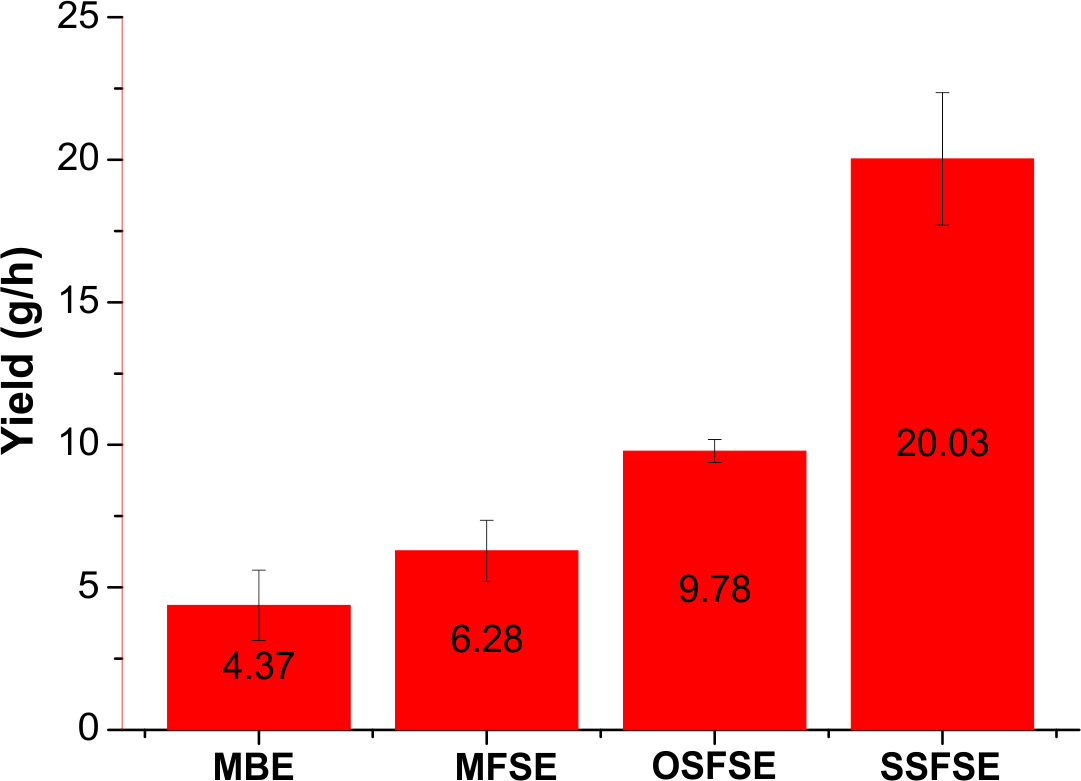
Yields of the PAN nanofibers produced using the different setups.

Figure 12 illustrates the yields of the PAN nanofibers fabricated by the different FSE devices. The yield of the MBE device is 4.37 g/h. In our previous work [23], the same device was used to prepare silk fibroin (SF) nanofibers with a yield of 3.1 g/h using a voltage of 50 kV. This finding is explained by the better spinnability of PAN compared to SF. When the structure of the reservoir is modified, the electric field distribution in the FSE process changes, and the nanofiber yield varies even when using the same voltage, working distance and spinning solution. As shown in Figure 12, the yield of the SSFSE method is the highest, followed by the OSFSE, MFSE and MBE methods. This might be because the bubble generation wastes energy possibly decelerating the charged jets during the MBE process resulting in a lower yield of PAN nanofibers. For the MFSE, OSFSE, and SSFSE methods the yields vary with the change of the electric field intensity. The distributions of the electric field intensities displayed in Figure 7 and Table 3 are in good agreement with the experimental results.

## Conclusion

In this paper, we presented four different self-made FSE devices, namely the MBE, MFSE, OSFSE and SSFSE devices, designed to obtain high-quality PAN nanofibers in large quantities. Using the Maxwell 3D software, the spinning mechanisms of the four FSE devices were studied by simulation of the electric field distribution, which is very import in the FSE process. The simulation results showed that the electric field distribution of the MBE device is the most nonuniform, and the electric field intensities inside the MFSE device are the smallest. The SSFSE device showed the best spinning performance owing to the uniform distribution of the electric field inside the device, as indicated by the ordered electric field vectors, and the largest resulting electric field intensity.

Furthermore, the impact of the device design on the morphology and the yield of nanofibers was experimentally investigated. We found that the average diameter of the nanofibers prepared by the MFSE method was the largest. The size distribution of the nanofibers produced by the MBE device was the least uniform. In contrast, the size distributions of the nanofiber generated by the of MFSE and SSFSE devices were more uniform. These experimental data are in accordance with the results of the simulations and the theoretical analysis. Our results show that the SSFSE is the best performing FSE device since it provides the highest quality and the maximum yield of nanofibers. Its yield could reach 20.03 g/h at an applied voltage of 40 kV. Compared with the performance of other spinning devices reported in the literature [13,17,20], the SSFSE device is highly suitable to produce large quantities of high-quality nanofibers at a low working voltage.
